# Supplementary material for: Otilonium Bromide Prevents Cholinergic Changes in the Distal Colon Induced by Chronic Water Avoidance Stress, a Rat Model of Irritable Bowel Syndrome
Source: Int J Mol Sci. 2023 Apr 18;24(8):7440. doi: 10.3390/ijms24087440 (PMC10139220; doi:10.3390/ijms24087440)
Supplement: Supplementary file 1 [file ijms-24-07440-s001.zip › ijms-2333707-supplementary.pdf]

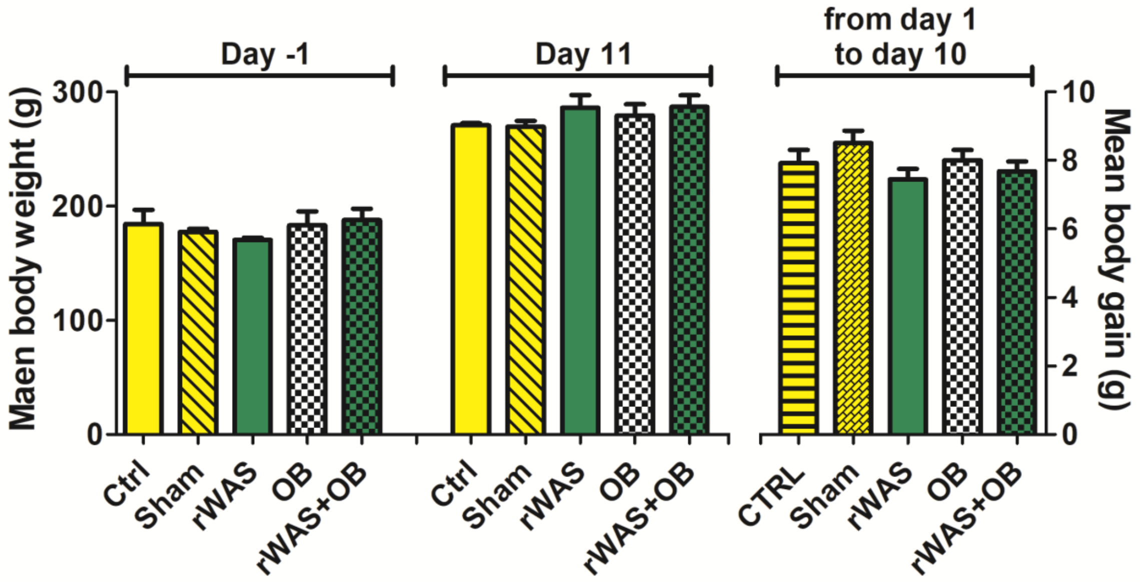

Figure S1. Time-course of weight gain. All animal groups had a similar weight at day -1 (left columns) and at day 11 (central columns) and the daily weight gain during the 10 days of stress application was comparable among the groups (right columns). One-way ANOVA followed by Newman-Keuls post-test, (n=7).

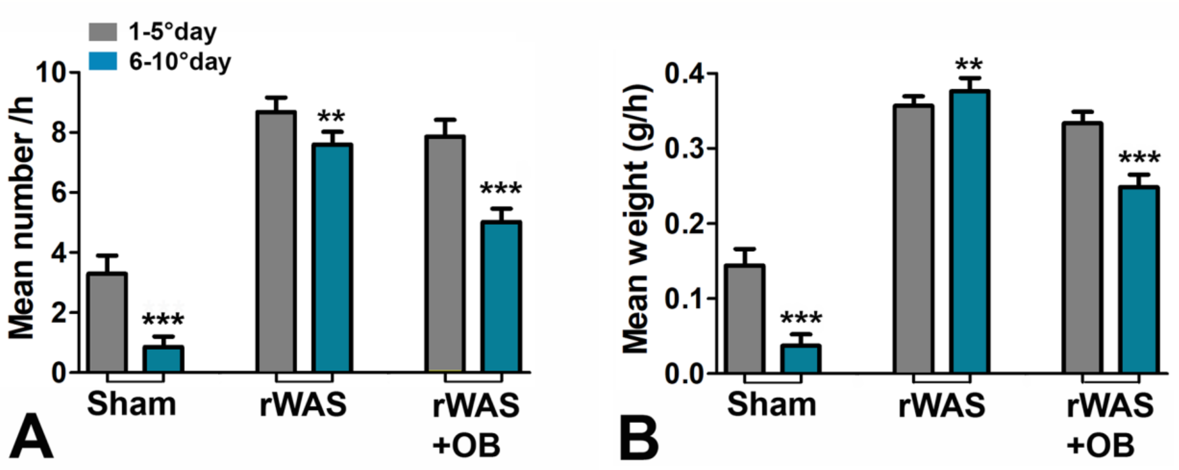

Figure S2. Fecal pellet production is expressed as mean number (A) and mean weight (B) per hour. In the rWAS both parameters were significantly higher compared to the Sham all along the entire period of stress application (columns grey and blue). In the rWAS+OB, the fecal pellet production decrease significantly in the second half period of stress exposition (blue columns) of treatment compared to the rWAS and similarly to the Sham (blue columns vs grey columns). Paired two-tailed Student t test, Sham; rWAS+OB ***$p<0.0001$ compared to the first 5 days of each group. One-way ANOVA followed by Newman-Keuls post-test, **$p<0.005$ compared to all the other groups. (n=7).
